# Synthesis, Spectroscopic and Semiempirical Studies of New Quaternary Alkylammonium Conjugates of Sterols

**DOI:** 10.3390/molecules181214961

**Published:** 2013-12-05

**Authors:** Bogumił Brycki, Hanna Koenig, Iwona Kowalczyk, Tomasz Pospieszny

**Affiliations:** Laboratory of Microbiocide Chemistry, Faculty of Chemistry, Adam Mickiewicz University, Grunwaldzka 6, Poznań 60-780, Poland; E-Mails: koenig@amu.edu.pl (H.K.); iwkow@amu.edu.pl (I.K.)

**Keywords:** sterols, quaternary alkylammonium salt, conjugates, Prediction of Activity Spectra for Substances, PM5 calculations

## Abstract

New quaternary alkylammonium conjugates of steroids were obtained by two step reaction of sterols (ergosterol, cholesterol, dihydrocholesterol) with bromoacetic acid bromide, followed by bimolecular nucleophilic substitution with a long chain tertiary alkylamine. The structures of products were confirmed by spectral (^1^H-NMR, ^13^C-NMR, and FT-IR) analysis, mass spectrometry and PM5 semiempirical methods. The pharmacotherapeutic potential of synthesized compounds has been estimated on the basis of Prediction of Activity Spectra for Substances (PASS).

## 1. Introduction

**Figure 1:**
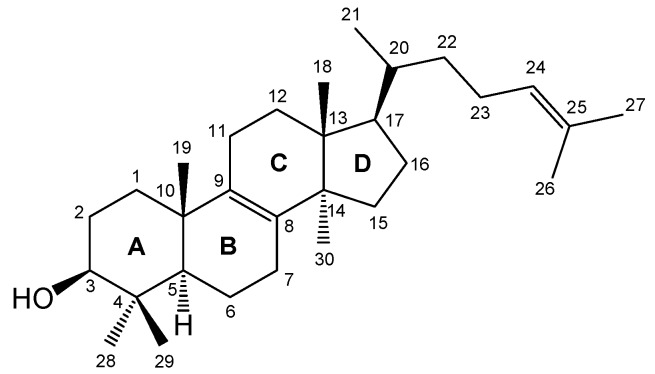
The structure, stereochemistry and numbering of lanosterol.

The steroids are modified triterpenoids with the tetracyclic ring system of lanosterol. However, the compounds do not have methyl groups at the C(4) and C(14) position and they have differently modified side chains [1,2] (Figure 1). The compounds of this type are of natural origin and play important biological functions in plant and animal cells. They are also the main sex hormones in mammals (e.g., testosterone and estrogens) and plants (e.g., brassinosteroids). They also regulate metabolism (e.g., glycocholic and taurocholic acid or vitamin D) and are important cardioactive glycosides (e.g., digoxin, gitoxin and scillaren A) [3,4,5,6].

Exceptionally interesting group of steroids are the sterols, e.g., cholesterol, cholestanol, ergosterol and stigmasterol [7,8,9]. Sterols are crystalline compounds which have a secondary hydroxyl group in the C(3) position of the steroid skeleton, one or two double bonds and differently modified side chains. Rings A/B of the steroid skeleton may have *trans* geometry (the allo series) or *cis* (the normal series). Sterols have a hydroxy group in the average plane of the ring, and can form a number of β-sterols [10,11].

**Figure 2:**
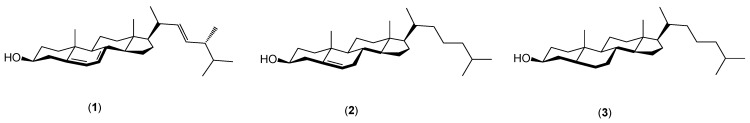
The structure of ergosterol (**1**), cholesterol (**2**) and cholestanol (**3**).

One of the most important sterol is ergosterol (**1**, provitamin D_2_), which performs analogous functions like cholesterol (**2**). but in the cells of fungi (Figure 2). Ergosterol is vital for fungal survival. It serves two purposes: a bulk membrane function and a vigorous function [1,2]. Furthermore ergosterol is a biological precursor to vitamin D_2_ [12,13]. Another important compound of this group is cholesterol (and its metabolite cholestanol (**3**)), which is fundamental component of the cell membranes of animal cells. Cholesterol in the ester form stabilizes and stiffens a protein–lipid membrane. Cholesterol in mammals regulates the cell membrane’s permeability and fluidity, growth rate and membrane-bound enzyme activity.

Modifications of functional groups in the molecules of sterols such as cholesterol or ergosterol provide compounds with high pharmacological activity. Connecting steroid compound molecules with natural products such as pyrimidines, purines, alkaloids or polyamines allows one to obtain new compounds with high biological activity as well as complexing or gelator agents. All compounds of this type may be classified as steroid conjugates [14].

Quaternary alkylammonium salts are very wide class of compounds which have many applications. Some of them are used as antiseptics and preservation agents [15]. It is proved that the derivatives which contain from 8 to 14 carbon atoms in the alkyl chain group show the greatest biocidal activity [16,17,18]. The mechanism of biocidal activity of quaternary alkylammonium salts is based on adsorption of the alkylammonium cation on the bacterial cell surface, diffusion through the cell wall and then binding and disruption of the cytoplasmatic membrane. Damage of the membrane results in a release of potassium ions and other cytoplasmatic constituents, finally leading to cell death [19,20,21,22,23,24]. A frequently used microbiocide, especially in sublethal concentrations, can result in an increasing resistance of microorganisms. One of the ways to overcome this serious negative side effect is the periodic application of new microbiocides with modified structures.

In recent years the number of applications of quaternary ammonium salts has increased constantly. They are used as biocides [19,20,21,22,23,24] and phase-transfer catalysts, especially in enantioselective reactions [25,26,27,28,29,30,31]. Some quaternary ammonium salts exist as ionic liquids, which can be used as “green solvents” [32,33,34] and electrolytes for liquid batteries [35,36]. Thus, the connection of plant sterols and long-chain amines or polyamines to form quaternary alkylammonium salts appears unusually interesting [37,38,39].

This work reports the synthesis and physicochemical properties of new quaternary alkylammonium conjugates of ergosteryl 3β-bromoacetate (**4**), cholesteryl 3β-bromoacetate (**5**) and dihydrocholesteryl 3β-bromoacetate (**6**) with *N*,*N*-dimethyl-*N*-octylamine (**7**, **11**, **15**), *N*,*N*-dimethyl-*N*-decylamine (**8**, **12**, **16**), *N*,*N*-dimethyl-*N*-dodecylamine (**9**, **13**, **17**), *N*,*N*-dimethyl-*N*-tetradecylamine (**10**, **14**, **18**) in acetonitrile. The potential pharmacological activities of the synthesized compounds have been studied using a computer-aided drug discovery approach with the *in silico* Prediction of Activity Spectra for Substances (PASSs) program. It is based on a robust analysis of the structure–activity relationships in a heterogeneous training set currently including about 60,000 biologically active compounds from different chemical series with about 4,500 types of biological activities. Since only the structural formula of the chemical compound is necessary to obtain a PASS prediction, this approach can be used at the earliest stages of investigation. There are many examples of the successful use of the PASS approach leading to new pharmacological agents [40,41,42,43,44]. The PASS software is useful for the study of biological activity of secondary metabolites. We have selected the types of activities that were predicted for a potential compound with the highest probability (focal activities). If predicted activity (PA) > 0.7, the substance is very likely to exhibit experimental activity and the chance of the substance being the analogue of a known pharmaceutical agent is also high. If 0.5 < PA < 0.7, the substance is unlikely to exhibit the activity in experiment, the probability is less, and the substance is unlike any known pharmaceutical agent.

## 2. Results and Discussion

**Scheme 1:**
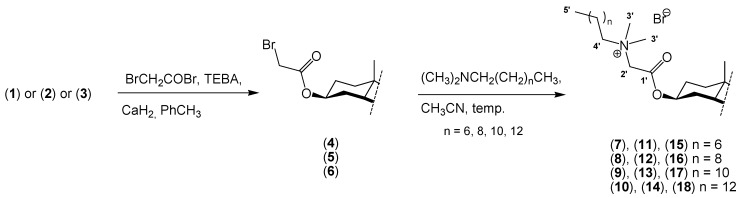
Synthesis of quaternary alkylammonium conjugates **7**–**18** of sterols **1**–**3**.

The new quaternary alkylammonium conjugates of steroids were obtained by reaction of sterols (ergosterol, cholesterol, dihydrocholesterol) with bromoacetic acid bromide to give intermediates **4**–**6**. The 3β-bromoacetates of sterols were prepared according to the literature procedures [45]. The structure of ergosteryl 3β-bromoacetate (**4**) was confirmed by ^1^H-NMR, ^13^C-NMR, and FT-IR analysis, as well as ESI-MS. The syntheses of conjugates **7**–**18** are shown in Scheme 1.

The structures of all synthesized compounds were determined from their ^1^H- and ^13^C-NMR, FT-IR and ESI-MS spectra. Moreover, PM5 calculations were performed on all compounds [46,47,48]. Additionally, analyses of the biological prediction activity spectra for the new esters prepared herein are good examples of *in silico* studies of chemical compounds.

We also selected the types of activity that were predicted for a potential compound with the highest probability (focal activities, Table 1). According to these data the most frequently predicted types of biological activity are: cholesterol antagonist, antihypercholesterolemic, adenomatous polyposis treatment and glyceryl-ether monooxygenase, acylcarnitine hydrolase, alcohol *O*-acetyltransferase, oxidoreductase, prostaglandin-E2 9-reductase, alkylacetylglycerophosphatase, alkenylglycerophospho- choline hydrolase or dextranase inhibitors, respectively.

**Table 1:** Probability “to be Active” (PA) values for predicted biological activity of compounds **7**–**18**.

Focal predicted activity (PA > 0.80)	Conjugates of		
	Ergosterol (1)	Cholesterol (2)	Cholestanol (3)
Cholesterol antagonist	0.880	0.904	0.873
Glyceryl-ether monooxygenase inhibitor	0.889	0.918	0.946
Antihypercholesterolemic	0.907	0.866	-
Acylcarnitine hydrolase inhibitor	-	0.873	0.967
Alcohol *O*-acetyltransferase inhibitor	0.911	-	-
Oxidoreductase inhibitor	0.881	-	-
Prostaglandin-E2 9-reductase inhibitor	-	0.857	-
Alkylacetylglycerophosphatase inhibitor	-	-	0.916
Alkenylglycerophosphocholine hydrolase inhibitor	-	-	0.899
Adenomatous polyposis treatment	-	-	0.825
Dextranase inhibitor	-	-	0.822

**Figure 3:**
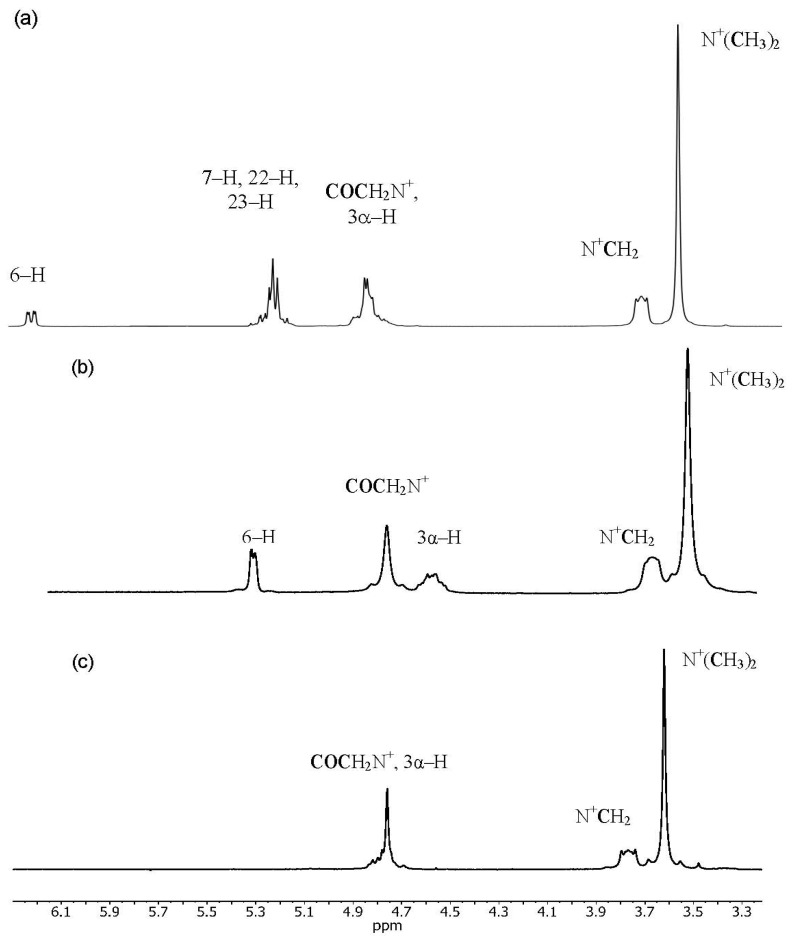
.^1^H-NMR spectra in the 6.2–3.3 ppm region showing the most characteristic signals of conjugates **9** (**a**), **13** (**b**) and **17** (**c**).

The ^1^H-NMR spectra of compounds **7**–**18** showed characteristic multiplets in the 4.90–4.64 ppm range assigned to the C3α–H protons of the sterol skeleton (Figure 3). Characteristic hydrogen singlets ranging from 0.68–0.65 ppm assigned to CH_3_–18. The second sets of singlets ranging from 1.02–1.00 ppm and 0.82 ppm were assigned to CH_3_–19 for **7**–**14** and **15**–**18**, respectively. The characteristic doublets of CH_3_–21 are at 1.04 ppm and 0.93–0.90 in the conjugates **7**–**10** and **11**–**18**, respectively. Overlapping multiplets appear in the 0.91–0.78 ppm range for CH_3_–26 and CH_3_–27 of the ergosterol- substituted derivatives. The ^1^H-NMR spectra of **11**–**18** showed a doublet at 0.86–0.85 ppm for the protons of the C(26) and C(27) methyl groups. For compounds **7**–**10** a doublet appears in the 0.93–0.91 ppm range assigned to CH_3_–28.

The ^1^H-NMR spectra of **7**–**18** showed a signal in the range 4.90–4.72 ppm for the protons of the COCH_2_N^+^ group. The signals for six methyl protons of the N^+^(CH_3_)_2_ and two methylene protons of the N^+^CH_2_ occurred as singlets and triplets in the 3.66–3.61 and 3.81–3.76 ppm range, respectively.

**Figure 4:**
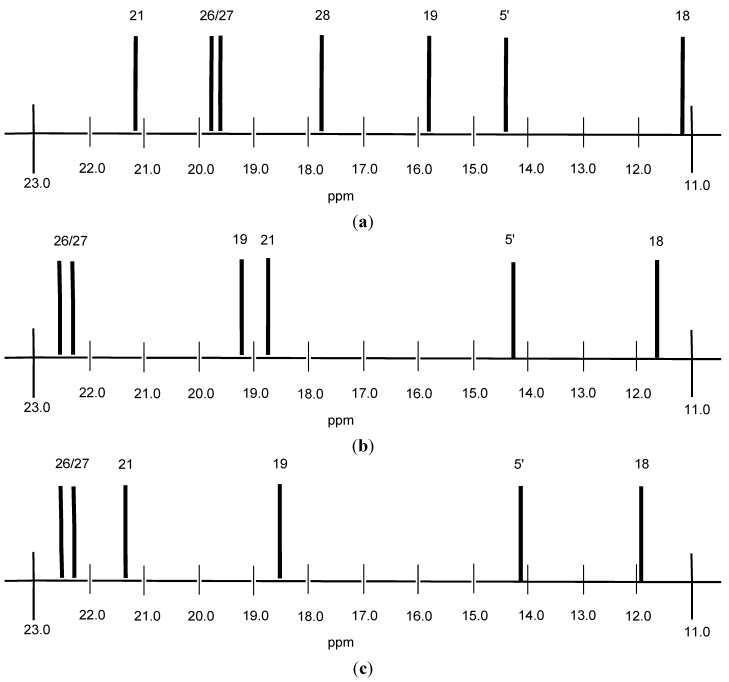
Analytical differences of CH_3_ groups of conjugates **9** (**a**), **13** (**b**) and **17** (**c**) in the corresponding ^13^C-NMR spectra.

The ^13^C-NMR spectra of compounds **7**–**18** show characteristic signals at 12.0–11.2 ppm and 21.2–21.0 ppm (**7**–**10**, **15**–**18**) or 18.1 ppm (**11**–**14**), which are assigned to CH_3_–18 and CH_3_–21, respectively. The carbons of the CH_3_–19 group gave signals in the ranges 16.1-15.8 ppm, 18.2 ppm and 18.6 ppm for **7**–**10**, **11**–**14** as well as **15**–**18**, respectively. Analytical differences in the ^13^C-NMR spectra of CH_3_ groups are shown in Figure 4.

Two important signals for C(1')=O and C(3)–O were present at 164.2–164.0 ppm and 76.7–76.0 ppm, respectively. The spectra of all conjugates show two diagnostic signals associated with CH_2_ atoms in N^+^–CH_2_–CO and N^+^–CH_2_ groups. The carbon atoms in the first group are located at 64.8–64.5 ppm and the second group 61.5–61.1 ppm, respectively. The carbon atoms of N^+^(CH_3_)_2_ the unit resonate in the 52.1–51.8 ppm range.

**Figure 5:**
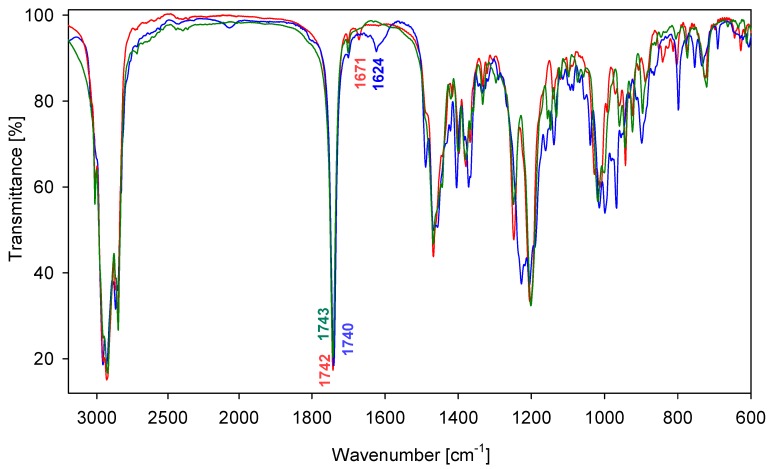
FT-IR spectra of conjugates **9** (blue), **13** (red) and **17** (green) in the 3,000–600 cm^−1^ region.

The solid-state IR spectra of representative conjugates **9**, **13** and **17** are shown in Figure 5. The intense bands in the 1,743–1,740 cm^−1^ region are due to the carbonyl group *ν*(C=O) stretching vibrations. Further coupling have little or no effect on the vibration of the carbonyl group. Moreover strong characteristic bands in the 1,248–1,227 cm^−1^ region are present, which are assigned to the *ν*(C–O). The *ν*(C=C) stretching vibration band of compounds **9** and **13** occurs at 1,670 cm^−^^1^and 1,624 cm^−1^ respectively, while the band is absent in compound **17**. The conjugated C=C bond stretching vibration shifts toward lower frequencies.

**Figure 6:**
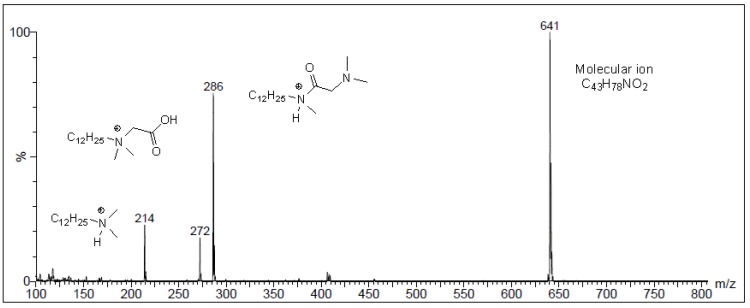
ESI-MS spectrum of conjugate **13**.

The ESI-MS spectra were recorded in methanol. In all cases, the molecular ion [M]^+^ is present, which is associated with the presence of a quaternary ammonium ion. In Figure 6 we present the ESI-MS spectrum of conjugate **13**. In the spectrum of this conjugate, the [M]^+^ molecular ion peak is observed at *m*/*z* 641 (100%). Elimination of the steroid skeleton, rearrangement of a hydrogen atom from the cholesteryl part to amine chain and simple cleavage of the C(1')Osp^3^–C(3)sp^3^ bond from the molecular ion of **13** gave the fragmenty ion [C_12_H_25_–N(CH_3_)_2_–CH_2_–CO_2_H]^+^ at *m*/*z* 272. The rearrangement of the quaternary alkylammonium chain connected to the amine chain part gave the [C_12_H_25_–NH(CH_3_)–CO–CH_2_–N(CH_3_)_2_]^+^ fragment ion at *m*/*z* 286 (75%). The cleavage of N(sp^3^)–C(sp^3^) bond leads to the formation of the [C_12_H_25_–N(CH_3_)_2_H]^+^ fragment ion situated at *m*/*z* 214.

**Figure 7:**
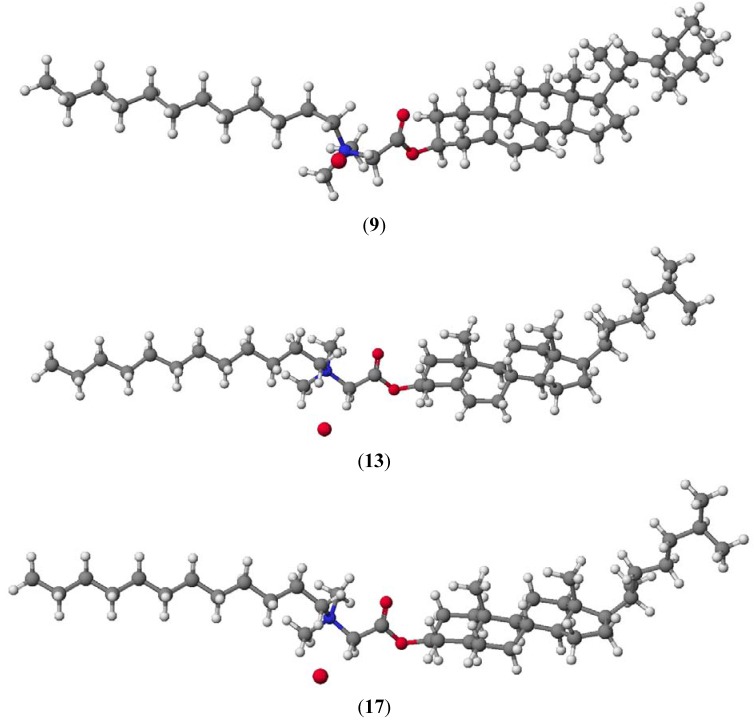
Molecular models of representative compounds (**9**), (**13**) and (**17**) calculated by PM5 method.

PM5 semiempirical calculations were performed using the WinMopac 2003 program. The final heat of formation (HOF) for the sterols **1**–**3** and conjugates **7**–**18** is presented in Table 2. Representative compounds **9**, **13** and **17** are shown in Figure 7. 

**Table 2:** Heat of formation (HOF) [kcal/mol] of sterols (**1**–**3**) and conjugates (**7**–**18**).

Compound	Heat of formation [kcal/mol]	ΔHOF [kcal/mol]
**1**	−97.1208	-
**2**	−140.1058	-
**3**	−162.7945	-
**7**	−166.1127	−68.9919
**8**	−177.2208	−80.1000
**9**	−185.2745	−88.1537
**10**	−199.4745	−102.3537
**11**	−209.1057	−68.9999
**12**	−220.2465	−80.1407
**13**	−231.3873	−91.2815
**14**	−242.5909	−102.4851
**15**	−231.7232	−68.9287
**16**	−242.8998	−80.1053
**17**	−253.9813	−91.1868
**18**	−265.2097	−102.4152

ΔHOF = HOF_conjugates (**7**–**18**)_ − HOF_sterols (**1**–**3**)_.

The lowest HOF value is observed for cholestanol (**3**) and its derivatives **15**–**18** where there are no double bonds to stabilize the molecules and hinder their reactivity. In derivatives **4**–**5** and **7**–**14** where double bonds are present increasing HOF values are observed. Furthermore, it was also observed that the extension of the hydrocarbon chain lowers the HOF values. 

**Figure 8:**
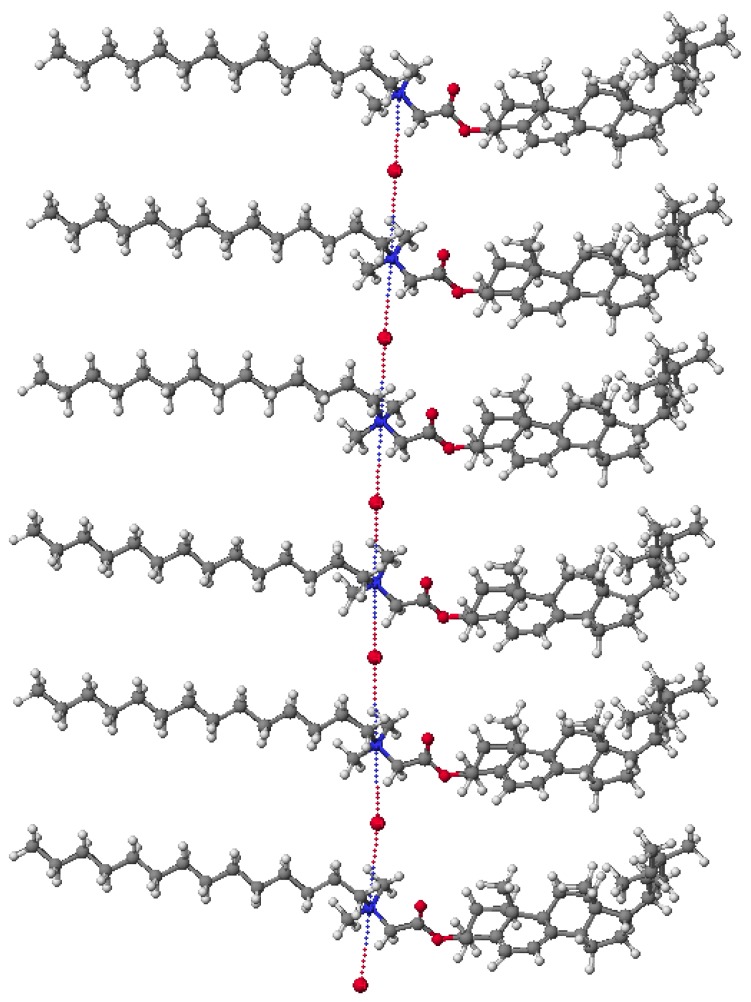
Molecular models of conjugates (**10**) calculated by PM5 method.

This fact can be explained by the increase in the number of possible conformers. In turn the length of the hydrocarbon chain is not without significance for the antimicrobial activity of the obtained conjugates. The spatial arrangement and interaction of the conjugate **10** is shown in Figure 8. The final heat of formation is −1249.429 kcal/mol and the distances between the quaternary nitrogen and the anion bromide are 4.19 Å. Compensation charges occur only through intermolecular electrostatic interaction. This is a very good confirmation of the conclusion that interactions reduce HOF.

## 3. Experimental

### 3.1. General

The NMR spectra were measured with a Spectrometer NMR Varian Mercury 300 (Oxford, UK), operating at 300.07 MHz and 75.4614 MHz for ^1^H and ^13^C, respectively. Typical conditions for the proton spectra were: pulse width 32°, acquisition time 5 s, FT size 32 K and digital resolution 0.3 Hz per point, and for the carbon spectra pulse width 60°, FT size 60 K and digital resolution 0.6 Hz per point, the number of scans varied from 1200 to 10,000 per spectrum. The ^13^C and ^1^H chemical shifts were measured in CDCl_3_ relative to an internal standard of TMS. Infrared spectra were recorded in the KBr pellets using a FT-IR Bruker IFS 66 spectrometer (Karlsruhe, Germany). The ESI (electron spray ionization) mass spectra were recorded on a Waters/Micromass (Manchester, UK) ZQ mass spectrometer equipped with a Harvard Apparatus (Saint Laurent, Canada), syringe pump. The sample solutions were prepared in methanol at the concentration of approximately 10^−5^ M. The standard ESI-MS mass spectra were recorded at the cone voltage 30 V.

### 3.2. Synthesis: Typical Procedure for the Synthesis of Quaternary Ammonium Conjugates of Sterols

Ergosteryl 3β-bromoacetate (cholesteryl 3β-bromoacetate or dihydrocholesteryl 3β-bromoacetate) (0.20 mmol) was dissolved in CH_3_CN (3 mL) under reflux. Then the appropriate amine (0.24 mmol) was added and the mixture heated under reflux for 2 h. The precipitate formed was filtered off and crystallized from CH_3_CN–EtOH (90:1), to give white solids.

*3β-Bromoacetate-ergosta-5,7,12-triene* (**4**): white solid (95%), m.p. 168–170 °C. ^1^H-NMR: δ_H_ 6.13 (dd, *J_1_* = 9.8, *J_2_* = 3.0 Hz, 1H, 6–H), 5.36–5.10 (m, 3H, 7–H, 22–H and 23–H), 4.96–4.68 (m, 1H, 3α–H), 3.81 (t, *J* = 3.0 Hz, 2H, COCH_2_Br), 1.04 (d, *J* = 6.6 Hz, 3H, CH_3_–21), 1.01 (s, 3H, CH_3_–19), 0.92 (d, *J* = 6.9 Hz, 3H, CH_3_–28), 0.91–0.81 (overlapping m, *J* = 6.7 Hz, 6H, CH_3_–26 and CH_3_–27), 0.67 (s, 3H, CH_3_–18). ^13^C-NMR: δ_C_ 166.80, 147.87, 135.35, 132,13, 128.51, 126.02, 118.05, 75.82, 55.95, 47.98, 44.47, 42.86, 40.73, 39.46, 38.88, 36.53, 34.81, 33.81, 33.08, 27.86, 27.13, 26.34, 24.97, 21.16, 19.98, 19.63, 18.25, 17.64, 15.82, 11.22. FT-IR (KBr) ν_max_: 3,003, 2,863, 1,778, 1,752, 1,609, 1,540, 1,377, 1,346, 1,278, 1,213, 1,055, 971. ESI-MS (*m*/*z*): 555 (40%) [C_30_H_45_O_2_Br+K]^+^, 539 (80%) [C_30_H_45_O_2_Br+Na]^+^, 524 (100%) [C_30_H_45_O_2_Br+Li]^+^, 517 (90%) [C_30_H_45_O_2_Br+H]^+^.

*N,N-dimethyl-(3β-acetate-ergosta-5,7,12-triene)-N-octylammonium bromide* (**7**): white solid (85%), m.p. 203–204 °C. ^1^H-NMR: δ_H_ 6.14 (dd, *J*_1_ = 9.8, *J*_2_ = 2.8 Hz, 1H, 6–H) 5.35–5.16 (m, 3H, 7–H, 22–H and 23–H ), 4.89–4.74 (m, 3H, COCH_2_N^+^ and 3α–H), 3.80 (t, *J* = 9.0 Hz, 2H, N^+^CH_2_), 3.65 (s, 6H, N^+^(CH_3_)_2_), 1.04 (d, *J* = 6.5 Hz, 3H, CH_3_–21), 1.00 (s, 3H, CH_3_–19), 0.93 (d, *J* = 6.9 Hz, 3H, CH_3_–28), 0.88-0.82 (overlapping m, 9H, CH_3_–26, CH_3_–27 and CH_3_–5'), 0.65 (s, 3H, CH_3_–18). ^13^C-NMR: δ_C_ 164.24, 148.21, 135.54, 132.20, 128.16, 123.46, 118.26, 76.72, 64.78, 61.35, 57.15, 52.06, 48.02, 44.87, 43.59, 42.91, 40.91, 38.97, 36.77, 36.52, 35.04, 33.96, 33.17, 31.69, 29.13, 29.06, 27.55, 26.18, 25.17, 22.99, 22.65, 21.90, 21.26, 21.08, 20.05, 19.73, 17.74, 15.91, 14.14, 11.34. FT-IR (KBr) ν_max_: 2,957, 2,930, 2,868, 1,740, 1,623, 1,489, 1,467, 1,404, 1,371, 1,227, 1,205, 1,138, 1,014, 999. ESI-MS (*m*/*z*): 754 (100%) [C_40_H_68_NO_2_Br_2_]^–^, 595 (100%) [C_40_H_68_NO_2_]^+^, 482 (20%) [C_32_H_51_NO_2_+H]^+^.

*N,N-dimethyl-(3β-acetate-ergosta-5,7,12-triene)-N-decylammonium bromide* (**8**): white solid (95%), m.p. 186–187 °C. ^1^H-NMR: δ_H_ 6.13 (d, *J* = 6.9 Hz, 1H), 5.28–5.12 (m, 3H, 7–H, 22–H, 23–H ), 4.88–4.74 (m, 3H, COCH_2_N^+^ and 3α–H), 3.76 (t, *J* = 6.0 Hz, 2H, N^+^CH_2_), 3.61 (s, 6H, N^+^(CH_3_)_2_), 1.04 (d, *J* = 6.5 Hz, 3H, CH_3_–21), 1.01 (s, 3H, CH_3_–19), 0.91 (d, *J* = 6,9 Hz, 3H, CH_3_–28), 0.88–0.78 (overlapping m, 9H, CH_3_–26, CH_3_–27 and CH_3_–5'), 0.65 (s, 3H, CH_3_–18). ^13^C-NMR: δ_C_ 164.05, 148.06, 135.43, 131.94, 128.01, 120.80, 116.14, 76.00, 64.61, 61.20, 55.62, 54.46, 51.92, 51.87, 45.91, 42.75, 40.38, 38.90, 37.66, 36.96, 36.29, 33.01, 31.78, 29.35, 29.28, 29.19, 29.05, 28.21, 27.85, 26.04, 22.91, 22.85, 22.60, 21.05, 20.95, 19.90, 19.59, 17.55, 16.07, 14.06, 12.00. FT-IR (KBr) ν_max_: 2,956, 2,927, 2,853, 1,742, 1,634, 1,458, 1,368, 1,251, 1,206, 1,021, 968. ESI-MS (*m*/*z*): 782 (100%) [C_42_H_72_NO_2_Br_2_]^–^, 623 (100%) [C_42_H_72_NO_2_]^+^, 244 (85%) [C_14_H_30_NO_2_]^+^.

*N,N-dimethyl-(3β-acetate-ergosta-5,7,12-triene)-N-dodecylammonium bromide* (**9**): white solid (95%), m.p. 194–196 °C. ^1^H-NMR: δ_H_ 6.13 (dd, *J*_1_ = 9.8, *J*_2_ = 2.8 Hz, 1H, 6–H), 5.24–5.13 (m, 3H, 7–H, 22–H, 23–H ), 4.90–4.72 (m, 3H, COCH_2_N^+^, 3α–H), 3.80 (t, *J* = 8.06 Hz, 2H, N^+^CH_2_), 3.67 (s, 6H, N^+^(CH_3_)_2_), 1.04 (d, *J* = 6.6 Hz, 3H, CH_3_–21), 1.00 (s, 3H, CH_3_–19), 0.92 (d, *J* = 6.8 Hz, 3H, CH_3_–28), 0.91–0.78 (overlapping m, 9H, CH_3_–26, CH_3_–27 and CH_3_–5'), 0.65 (s, 3H, CH_3_–18). ^13^C-NMR: δ_C_ 164.06, 148.08, 135.25, 132.10, 128.00, 123.30, 118.11, 76.57, 64.62, 61.17, 55.89, 52.00, 47.87, 44.41, 43.45, 42.81, 39.41, 36.43, 35.52, 34.70, 33.03, 32.19, 31.85, 29.55, 29.40, 29.28, 29.04, 27.80, 27.15, 26.05, 24.93, 22.85, 22.63, 21.11, 19.93, 19.59, 19.36, 17.60, 15.76, 14.08, 11.19. FT-IR (KBr) ν_max_: 2,957, 2,926, 2,852, 1,741, 1,631, 1,467, 1,398, 1,248, 1,203, 1,012, 969. ESI-MS (*m*/*z*): 651 (100%) [C_44_H_76_NO_3_]^+^, 272 (50%) [C_16_H_34_NO_2_]^+^.

*N,N-dimethyl-(3β-acetate-ergosta-5,7,12-triene)-N-tetradecylammonium bromide* (**10**): white solid (92%), m.p. 198–200 °C. ^1^H-NMR: δ_H_ 6.14 (dd, *J*_1_ = 9.7, *J*_2_ = 2.8 Hz, 1H, 6–H), 5.36–5.17 (m, 3H, 7–H, 22–H, 23–H ) 4.86-4.79 (m, 3H, COCH_2_N^+^, 3α–H), 3.79 (t, *J* = 8.0 Hz, 2H, N^+^CH_2_), 3.65 (s, 6H, N^+^(CH_3_)_2_), 1.04 (d, *J* = 6.6 Hz, 3H, CH_3_–21), 1.00 (s, 3H, CH_3_–19), 0.92 (d, *J* = 12.1 Hz, 3H, CH_3_–28), 0.90–0.82 (m, 9H, CH_3_–26, CH_3_–27 and CH_3_–5'), 0.65 (s, 3H, CH_3_–18). ^13^C-NMR: δ_C_ 164.09, 148.07, 135.26, 132.10, 128.01, 123.30, 118.12, 76.67, 64.67, 61.23, 57.01, 55.90, 51.99, 47.87, 44.42, 43.45, 42.82, 40.76, 39.42, 38.84, 36.45, 35.53, 34.71, 33.04, 32.20, 31.88, 29.61, 29.41, 29.32, 29.28, 29.05, 27.81, 27.17, 26.06, 24.94, 22.87, 22.65, 21.12, 19.94, 19.61, 17.61, 15.76, 14.09, 11.20. FT-IR (KBr) ν_max_: 2,957, 2,852, 1,743, 1,635, 1,467, 1,371, 1,247, 1,205, 1,017, 973. ESI-MS (*m*/*z*): 839 (100%) [C_46_H_80_NO_2_Br_2_]^–^, 679 (100%) [C_46_H_80_NO_2_]^+^.

*N,N-dimethyl-(3β-acetate-cholest-5-ene)-N-octylammonium bromide* (**11**): white solid (99%), m.p. 210–211 °C. ^1^H-NMR: δ_H_ 5.40 (d, *J* = 4.8 Hz, 1H, 6–H), 4.85 (d, *J* = 3.6 Hz, 2H, COCH_2_N^+^), 4.71–4.65 (m, 1H, 3α–H), 3.80 (t, *J* = 8.1 Hz, 2H, N^+^CH_2_), 3.65 (s, 6H, N^+^(CH_3_)_2_), 2.35 (d, *J* = 6.3 Hz, 2H, 4–CH_2_), 1.01 (s, 3H, CH_3_–19), 0.92 (d, *J =* 6.5 Hz, 3H, CH_3_–21), 0.88 (t, *J =* 3.0Hz, 3H, CH_3_–5'), 0.86 (d, *J =* 1.3 Hz, 3H, CH_3_–26), 0.85 (d, *J =* 1.3 Hz, 3H, CH_3_–27), 0.68 (s, 3H, CH_3_–18). ^13^C-NMR: δ_C_ 164.01, 138.67, 123.45, 76.67, 64.60, 61.15, 56.58, 56.03, 51.80, 49.88, 42.22, 39.60, 39.44, 37.74, 36.74, 36.46, 36.10, 35.71, 31.82, 31.71, 31.55, 28.99, 28.92, 28.15, 27.95, 27.51, 26.02, 24.20, 23.75, 22.84, 22.77, 22.51, 20.95, 19.20, 18.65, 14.01, 11.78. FT-IR (KBr) ν_max_: 2,956, 2,934, 2,868, 1,741, 1,671, 1,488, 1,467, 1,378, 1,228, 1,211, 1,014, 996. ESI-MS (*m*/*z*): 744 (100%) [C_39_H_70_NO_2_Br_2_]^-^, 584 (100%) [C_39_H_70_NO_2_]^+^.

*N,N-dimethyl-(3β-acetate-cholest-5-ene)-N-decylammonium bromide* (**12**): white solid (82%), m.p. 210–212 °C. ^1^H-NMR: δ_H_ 5.39 (d, *J* = 4.3 Hz, 1H, 6–H), 4.85 (d, *J* = 3.6 Hz, 2H, COCH_2_N^+^), 4.72–4.64 (m, 1H, 3α–H), 3.80 (t, *J* = 8.1 Hz, 2H, N^+^CH_2_), 3.65 (s, 6H, N^+^(CH_3_)_2_), 2.35 (d, *J* = 6.1 Hz, 2H, 4–CH_2_), 1.01 (s, 3H, CH_3_-19), 0.92 (d, *J* = 6.7 Hz, 3H, CH_3_–21), 0.88 (t, *J* = 3.0 Hz, 3H, CH_3_–5'), 0.86 (d, *J* = 1.3 Hz, 3H, CH_3_–26), 0.85 (d, *J* = 1.3 Hz, 3H, CH_3_–27), 0.68 (s, 3H, CH_3_–18). ^13^C-NMR: δ_C_ 164.01, 138.67, 123.45, 76.68, 64.60, 61.15, 56.58, 56.04, 51.83, 49.88, 42.23, 39.60, 39.44, 37.75, 36.74, 36.46, 36.10, 35.71, 31.82, 31.78, 31.72, 29.34, 29.27, 29.19, 29.04, 28.15, 27.95, 27.51, 26.03, 24.20, 23.75, 22.84, 22.77, 22.61, 22.51, 20.95, 19.21, 18.65, 14.07, 11.78. . FT-IR (KBr) ν_max_: 2,953, 2,853, 1,742, 1,467, 1,379, 1,248, 1,200, 1,139, 1,026, 942. ESI-MS (*m/z*): 773 (100%) [C_41_H_74_NO_2_Br_2_]^–^, 613 (100%) [C_41_H_74_NO_2_]^+^. 

*N,N-dimethyl-(3β-acetate-cholest-5-ene)-N-dodecylammonium bromide* (**13**): white solid (92%), m.p. 213–214 °C. ^1^H-NMR: δ_H_ 5.39 (d, *J* = 4.4 Hz, 1H, 6–H), 4.86 (brs, 2H, COCH_2_N^+^), 4.70–4.67 (m, 1H, 3α–H), 3.80 (t, *J* = 12.1 Hz, 2H, N^+^CH_2_), 3.66 (s, 6H, N^+^(CH_3_)_2_), 2.35 (d, *J* = 6.9 Hz, 2H, 4–CH_2_), 1.02 (s, 3H, CH_3_–19), 0.92 (d, *J* = 6.7 Hz, 3H, CH_3_–21), 0.88 (t, *J* = 3.0 Hz, 3H, CH_3_–5'), 0.86 (d, *J* = 1.3 Hz, 3H, CH_3_–26), 0.85 (d, *J* = 1.3 Hz, 3H, CH_3_–27), 0.68 (s, 3H, CH_3_–18). ^13^C-NMR: δ_C_ 164.05, 138.70, 123.44, 76.57, 64.63, 61.22, 56.61, 56.08, 51.88, 49.93, 42.25, 39.63, 39.45, 37.77, 36.77, 36.48, 36.13, 35.72, 31.85, 31.75, 29.55, 29.40, 29.28, 29.04, 28.15, 27.95, 27.54, 26.05, 24.21, 23.77, 22.86, 22.76, 22.63, 22.50, 20.97, 19.21, 18.66, 14.07, 11.79. FT-IR (KBr) ν_max_: 2,954, 2,930, 2,851, 1,742, 1,700, 1,671, 1,468, 1,397, 1,378, 1,248, 1,204, 1,015, 943. ESI-MS (*m*/*z*): 800 (100%) [C_43_H_78_NO_2_Br_2_]^–^, 641 (100%) [C_43_H_78_NO_2_]^+^.

*N,N-dimethyl-(3β-acetate-cholest-5-ene)-N-tetradecylammonium bromide* (**14**): white solid (92%), m.p. 197–199 °C. ^1^H-NMR: δ_H_ 5.39 (d, *J* = 4.67 Hz, 1H, 6–H), 4.86 (brs, 2H, COCH_2_N^+^), 4.71–4.64 (m, 1H, 3α–H), 3.81 (brs, 2H, N^+^CH_2_), 3.66 (s, 6H, N^+^(CH_3_)_2_), 2.36 (d, *J* = 6.9 Hz, 2H, 4–CH_2_), 1.01 (s, 3H, CH_3_–19), 0.93 (d, *J* = 6.7 Hz, 3H, CH_3_–21), 0.88 (t, *J* = 3.0 Hz, 3H, CH_3_–5'), 0.86 (d, *J* = 1.3 Hz, 6H, CH_3_–26 and CH_3_–27), 0.68 (s, 3H, CH_3_–18). ^13^C-NMR: δ_C_ 164.04, 138.69, 123.43, 76.57, 64.60, 61.20, 56.60, 56.06, 51.88, 49.91, 42.24, 39.63, 39.44, 37.76, 36.75, 36.47, 36.11, 35.71, 31.87, 31.76, 29.60, 29.40, 29.27, 29.03, 28.15, 27.94, 27.52, 26.03, 24.21, 23.76, 22.84, 22.75, 22.64, 22.50, 20.96, 19.21, 18.65, 14.07, 11.79. FT-IR (KBr) ν_max_: 2,954, 2,927, 1,743, 1,467, 1,404, 1,379, 1,286, 1,247, 1,202, 1,175, 1,008, 945. ESI-MS (*m*/*z*): 828 (100%) [C_45_H_82_NO_2_Br_2_]^–^, 669 (100%) [C_45_H_82_NO_2_]^+^.

*N,N-dimethyl-(3β-acetate-5β-cholestan)-N-octylammonium bromide* (**15**): white solid (95%), m.p. 209–210 °C. ^1^H-NMR: δ_H_ 4.81–4.74 (m, 3H, 3α–H, COCH_2_N^+^), 3.78 (t, *J* = 8.1 Hz, 2H, N^+^CH_2_), 3.63 (s, 6H, N^+^(CH_3_)_2_), 0.91 (d, *J* = 6.6 Hz, 3H, CH_3_–21), 0.88 (t, *J* = 3.0 Hz, 3H, CH_3_–5'), 0.85 (d, *J* = 1.3 Hz, 6H, CH_3_–26 and CH_3_–27), 0.82 (s, 3H, CH_3_–19), 0.65 (s, 3H, CH_3_–18). ^13^C-NMR: δ_C_ 164.04 , 76.57, 64.83, 61.48, 56.33, 56.21, 54.09, 52.26 , 44.62, 42.52, 39.89, 39.45, 36.56, 36.11, 35.73, 35.37, 33.71, 31.87, 31.57, 29.02, 28.93, 28.48, 28.17, 27.95, 27.27, 26.10, 24.13, 23.78, 22.94, 22.76, 22.52, 21.15, 18.62, 14.01, 12.20, 12.01. FT-IR (KBr) ν_max_: 2,928, 2,851, 1,740, 1,488, 1,468, 1,405, 1,378, 1,228, 1,209, 1,001, 957, 927, 897. ESI-MS (*m*/*z*): 746 (100%) [C_39_H_72_NO_2_Br_2_]^–^, 587 (100%) [C_39_H_72_NO_2_]^+^.

*N,N-dimethyl-(3β-acetate-5β-cholestan)-N-decylammonium bromide* (**16**): white solid (93%), m.p. 209–210 °C. ^1^H-NMR: δ_H_ 4.86–4.74 (m, 3H, 3α-H and COCH_2_N^+^), 3.79 (t, *J* = 9.0 Hz, 2H, N^+^CH_2_), 3.65 (s, 6H, N^+^(CH_3_)_2_), 0.91 (d, *J* = 6.6 Hz, 3H, CH_3_–21), 0.88 (t, *J* = 3.0 Hz, 3H, CH_3_–5'), 0.85 (d, *J* = 1.3 Hz, 6H, CH_3_–26 and CH_3_–27), 0.82 (s, 3H, CH_3_–19), 0.65 (s, 3H, CH_3_–18). ^13^C-NMR: δ_C_ 164.07, 76.58, 64.64, 61.21 , 56.34, 56.22, 54.09, 51.90, 44.61, 42.53, 39.89, 39.46, 36.56, 36.11, 35.73, 35.36, 33.67, 31.87, 31.78, 29.34, 29.27, 29.19, 29.04, 28.47, 28.17, 27.96, 27.22, 26.04, 24.14, 23.78, 22.85, 22.76, 22.61, 22.51, 21.15, 18.62, 14.06, 12.19, 12.01. FT-IR (KBr) ν_max_: 2,954, 2,927, 2,853, 1,741, 1,467, 1,398, 1,248, 1,200, 1,147, 1,134, 1,019, 942. ESI-MS (*m*/*z*): 774 (100%) [C_41_H_76_NO_2_Br_2_]^–^, 615 (100%) [C_41_H_76_NO_2_]^+^.

*N,N-dimethyl-(3β-acetate-5β-cholestan)-N-dodecylammonium bromide* (**17**): white solid (92%), m.p. 210–212 °C. ^1^H-NMR: δ_H_ 4.82–4.76 (m, 3H, 3α–H, COCH_2_N^+^), 3.77 (t, *J* = 9.0 Hz, 2H, N^+^CH_2_), 3.62 (s, 6H, N^+^(CH_3_)_2_), 0.90 (d, *J* = 6.00 Hz, 3H, CH_3_–21), 0.88 (t, *J* = 3.0 Hz, 3H, CH_3_–5'), 0.85 (d, *J* = 1.4 Hz, 6H, CH_3_–26 and CH_3_–27), 0.82 (s, 3H, CH_3_–19), 0.65 (s, 3H, CH_3_–18). ^13^C-NMR: δ_C_ 164.00, 76.56, 64.77, 61.20, 56.32, 56.20, 54.08, 51.90, 44.59, 42.51, 39.87, 39.44, 36.54, 36.09, 35.71, 35.34, 33.65, 31.84, 29.52, 29.37, 29.30, 29.26, 29.23, 29.00, 28.45, 28.15, 27.93, 27.19, 26.00, 24.11, 23.75, 22.78, 22.73, 22.61, 22.48, 21.14, 18.60, 14.05, 12.16, 11.99. FT-IR (KBr) ν_max_: 2,927, 2,850, 1,741, 1,467, 1,397, 1,379, 1,333, 1,248, 1,199, 1,015, 1,000. ESI-MS (*m/z*): 802 (100%) [C_43_H_80_NO_2_Br_2_]^–^, 643 (20%) [C_43_H_80_NO_2_]^+^, 272 (100%) [C_16_H_34_NO_2_]^+^.

*N,N-dimethyl-(3β-acetate-5β-cholestan)-N-tetradecylammonium bromide* (**18**): white solid (91%), m.p. 195–196 °C. ^1^H-NMR: δ_H_ 4.80–4.75 (m, 3H, 3α–H, COCH_2_N^+^), 3.80(t, *J* = 8.1 Hz, 2H, N^+^CH_2_), 3.65 (s, 6H, N^+^(CH_3_)_2_), 0.91 (d, *J* = 6.6 Hz, 3H, CH_3_–21), 0.88 (t, *J* = 3.0 Hz, 3H, CH_3_–5'), 0.85 (d, *J* = 1.4 Hz, CH_3_–26 and CH_3_–27), 0.82 (s, 3H, CH_3_–19), 0.65 (s, 3H, CH_3_–18).^13^C-NMR: δ_C_ 164.04, 76.68, 64.54, 61.14, 56.30, 56.18, 54.05, 51.88, 44.58, 44.52, 42.49, 39.85, 39.43, 36.53, 36.08, 35.71, 35.33, 33.64, 31.86, 31.84, 29.64, 29.60, 29.54, 29.39, 29.31, 29.25, 29.01, 28.45, 28.15, 27.93, 27.19, 27.12, 26.4, 26.06, 24.11, 23.75, 22.81, 22.75, 22.63, 22.53, 22.49, 21.13, 18.59, 14.07, 12.17, 11.98. FT-IR (KBr) ν_max_: 2,954, 2,849, 1,742, 1,468, 1,399, 1,379, 1,333, 1,248, 1,201, 1,018, 959. ESI-MS (*m*/*z*): 831 (100%) [C_43_H_80_NO_2_Br_2_]^–^, 671 (100%) [C_45_H_84_NO_2_]^+^. 

## 4. Conclusions

In summary, twelve new quaternary ammonium conjugates of sterols **7**–**18** were prepared by the reactions in acetonitrile of ergosteryl 3β-bromoacetate, cholesteryl 3β-bromoacetate and dihydrocholesteryl 3β-bromoacetate, with *N*,*N*-dimethyl-*N*-octylamine, *N*,*N*-dimethyl-*N*-decylamine, *N*,*N*-dimethyl-*N*-dodecylamine and *N*,*N*-dimethyl-*N*-tetradecylamine. These new compounds were characterized by spectroscopic and molecular structure methods. These conjugates may find applications in molecular recognition and in pharmacology, especially as compounds with a high antimicrobial activity.
